# Biotechnological and Pharmacological Applications of Biotoxins and Other Bioactive Molecules from Dinoflagellates

**DOI:** 10.3390/md15120393

**Published:** 2017-12-20

**Authors:** Joana Assunção, A. Catarina Guedes, F. Xavier Malcata

**Affiliations:** 1 LEPABE—Laboratory of Process Engineering, Environment, Biotechnology and Energy, Rua Dr. Roberto Frias, s/n, P-4200-465 Porto, Portugal; joanaleonardoassuncao@gmail.com; 2 Interdisciplinary Centre of Marine and Environmental Research (CIIMAR), University of Porto, Terminal de Cruzeiros do Porto de Leixões, Avenida General Norton de Matos, s/n, P-4450-208 Matosinhos, Portugal; acatarinaguedes@gmail.com; 3 Department of Chemical Engineering, University of Porto, Rua Dr. Roberto Frias, s/n, P-4200-465 Porto, Portugal

**Keywords:** biotoxin, microalgae, pharmacological applications, microbial factories, therapeutical value

## Abstract

The long-lasting interest in bioactive molecules (namely toxins) produced by (microalga) dinoflagellates has risen in recent years. Exhibiting wide diversity and complexity, said compounds are well-recognized for their biological features, with great potential for use as pharmaceutical therapies and biological research probes. Unfortunately, provision of those compounds is still far from sufficient, especially in view of an increasing demand for preclinical testing. Despite the difficulties to establish dinoflagellate cultures and obtain reasonable productivities of such compounds, intensive research has permitted a number of advances in the field. This paper accordingly reviews the characteristics of some of the most important biotoxins (and other bioactive substances) produced by dinoflagellates. It also presents and discusses (to some length) the main advances pertaining to dinoflagellate production, from bench to large scale—with an emphasis on material published since the latest review available on the subject. Such advances encompass improvements in nutrient formulation and light supply as major operational conditions; they have permitted adaptation of classical designs, and aided the development of novel configurations for dinoflagellate growth—even though shearing-related issues remain a major challenge.

## 1. Introduction

During the latest few years, demand for new biocompounds, and its derivatives with biotechnological and pharmacological potential have experienced a remarkable increase [1]. This trend—to create/find innovative and competitive products through win-win approaches—has placed a considerable emphasis upon research on marine organisms, including microalgae and macroalgae [2]. Dinoflagellates, in particular, are a unique group of the former; they are unicellular planktonic microalgae, and a source of biotoxins affecting seafood safety—yet bearing a potential for human health at large. A recent boom has indeed been noticed, due to their unexpected applications as pharmacological drugs, and potential uses in the biology, biomedical and toxicological fields [3].

Commonly found in all types of ecosystems (marine, freshwater, benthic and even sea ice), this complex taxon is estimated to include 2000+ living species [4]. Half of them are photoautotrophs [5], whereas the remainder rely on mixotrophy or heterotrophy, as well as parasitic or symbiotic behaviors [3,6]. Ecologically speaking, dinoflagellates possessing photosynthetic pigments play a major role as primary producers in freshwater and marine habitats [7]. They can also be found in association with other marine organisms, e.g., sea anemones, protozoa, tissues of certain invertebrates, and stony corals [8].

Dinoflagellates have been reported as potent natural biotoxin producers, as many of these compounds are effective at far lower dosages than conventional chemical agents [9]. Their ability to synthesize toxic compounds, accompanied by sudden and booming growth in marine environments make them the major cause of harmful algal blooms (HABs). HABs cause discoloration on the sea surface, and are thus called ‘red tides’; 75–80% of toxic phytoplankton species therein are in fact dinoflagellates [10]. ‘Red tides’ may vary in color from common red to brown, yellow, green or blue, depending on the dominant species, concentration and depth [11]. Such high densities of dinoflagellates have been associated to aquatic faunal mortalities worldwide, since they can kill fish and/or shellfish—either directly via toxin production, or because their large numbers block animal gills and deplete available oxygen [12]. In addition, toxins accumulated in these organisms can be transferred to higher trophic levels through the food chain [9]. The negative impact on coastal areas is particularly notorious; it can not only disrupt the marine environment, but also disturb human economic activities (e.g., tourism, aquaculture, fisheries), and ultimately affect human health. How and why these natural phenomena occur remain to be fully understood, but weather and hydrographic conditions probably play a role [13].

According to the Taxonomic Reference List of Toxic Plankton Algae of Intergovernmental Oceanographic Commission (IOC), there are at least 95 dinoflagellates known to produce toxins (called phycotoxins), among 179 species of marine microalgae [14]. For some toxins, doses at the microgram per kilogram level are more than sufficient to kill [15]. It is not clear why some microalgal species produce biotoxins. Quilliam [16] claimed some of these second metabolites to be allellochemical agents against other species, aids in competing for a specific niche, defense against predation, and enzyme regulation or sexual response induction (feromones).

Several biotoxins produced by marine microalgae can traditionally be organized on the basis on their effects upon humans: paralytic shellfish poisoning (PSP), neurotoxic shellfish poisoning (NSP), diarrheic shellfish poisoning (DSP) and amnesic shellfish poisoning (ASP)—released by diatoms, azaspiracid poisoning (AZP), and ciguatera fish poisoning (CFP). Most such poisonings are caused by neurotoxins, which exhibit highly specific effects upon the nervous system in birds, animals or even humans upon ingestion of contaminated shellfish [9]. Human clinical effects/symptoms have been revised elsewhere [9,17].

Despite the above unfavorable aspects, the unique structure and diverse functionality of dinoflagellate biotoxins make them valuable and quite interesting compounds. Several toxicological and biological studies, entailing mixed and axenic cultures, have unfolded the potential of dinoflagellate-derived compounds (including biotoxins) as promising pharmacological effectors and/or biological investigation probes. 

This review will focus on the pharmacological and biotechnological potential specifically of dinoflagellate-originated biotoxins, but will also briefly cover other important bioactive compounds produced thereby for the sake of completeness. It will also provide a brief overview of efforts (in terms of bioreactors and operational conditions) to improve their production, and discuss a few issues that still need improvement in attempts to attain higher dinoflagellate biomass and toxin productivities.

## 2. Characterization of Main Dinoflagellate Bioactive (Potential) Applications

Dinoflagellates are able to produce bioactive compounds with distinctive chemical structures, and a wide range of functional groups and toxicological and biological features; macrolides, cyclic polyethers, spirolides and purine alkaloids are but examples of such categories [18]. Due to their disparate functional structures, said biocompounds form a heterogeneous group that may strongly affect a variety of biological receptors and metabolic processes [10]. Hence, they may find application in human or veterinary medicine—in view of their wide range of potential pharmacological activities, i.e., in analgesic, antitumor, anticholesterol, cytotoxic, anti-infective, immuno-suppressive and/or neurological disease therapeutics [3]. Furthermore, other interesting dinoflagellate-derived compounds, such as pigments (e.g., peridinin), fatty acids (e.g., PUFAs) or polysaccharides, have shown noteworthy evidence for extra health benefits as nutraceuticals, prevention of development and anti-proliferation of tumor cells, and anti-inflammatory and antiviral activities [2,19,20].

Due to their biological potential—and despite several difficulties in getting the minimum amounts of biotoxins for testing, several studies and patents encompassing applications of dinoflagellates, directly associated to biotoxins, have been published or filed (Table 1). 

The next sub-sections entail an overview of the most important biotoxins, complemented later by a brief reference to selected bioactives (i.e., gambieric acid, goniodomin, ampidinolide and ampidinol)—regarding their mode of action and biological potential, toward pharmaceutical and biotechnological applications. 

### 2.1. Saxitoxin (and Analogues)

Saxitoxin (STX) and ca. six dozen naturally occurring analogues (such as gonyautoxins and neosaxitoxin) are produced mainly by marine dinoflagellates belonging to genera *Alexandrium* (e.g., *A. minutum*, *A. tamarense*, *A. catenella*), *Gymnodinium* (*G. catenatum*) and *Pyrodinium* (*P. bahamense*). However, other sources of STX-group toxins were identified—as is the case of cyanobacteria, including *Anabaena*, *Cylindrospermopsis*, *Aphanizomenon*, *Planktothrix* and *Lyngbya* genera [21]. 

**Figure 1:**
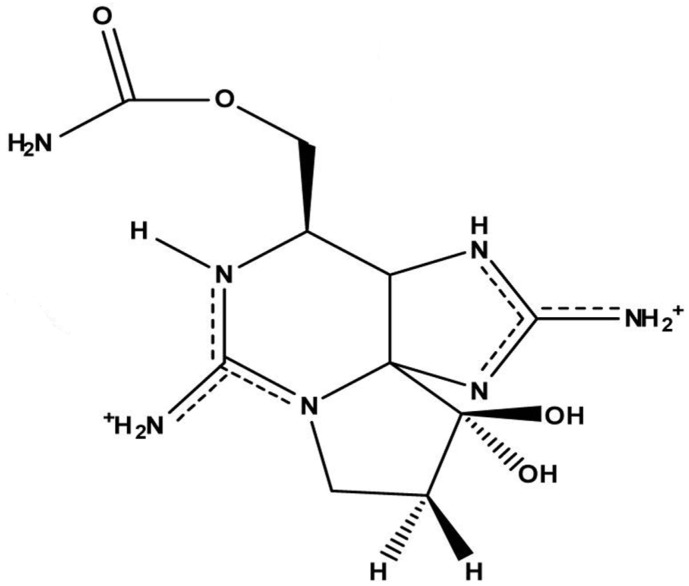
Chemical structure of saxitoxin (STX) (adapted from [21]).

STX is an alkaloid belonging to a group of marine natural products containing guanidine groups as main structural components. STX is composed of a 3,4-propinoperhydropurine tricyclic system (Figure 1), and the presence of two guanidine groups makes this molecule highly polar [21,22,23]. According to the specific functional group, there are carbamonyl (e.g., STX, neosaxitoxin, gonyautoxin 1 and 4 GTX1 and GTX4), decarbamoyl (e.g., GTX2 and GTX3) and *N*-sulfocarbamoyl (i.e., GTX5 and GTX6) saxitoxins. Another group comprises hydroxylated saxitoxins [24]. The corresponding chemical substituent in the main structure is a clue to its toxic potency (carbamoyl > decarbamoyl > *N*-sulfocarbamoyl) in model organisms (i.e., mouse) [24,25]. 

This group of biotoxins act as highly selective sodium channel blockers, thus preventing the influx flow of Na^+^ ions and compromising generation of action potentials [21,26]. Since STXs and their analogues make neurons and muscle cells lose their ability to transmit electrical impulses [27], they have a therapeutic potential as anesthetic agents. They may indeed reduce, or even block pain sensation, decrease muscle spasm, induce muscle relaxation and reduce wrinkles. Despite its potential applications, human clinical trials pose an obstacle—since toxicity often persists [21]. Several studies suggest that interaction with binding site 1 of voltage-gated Na^+^ channels (VGSCs) can induce prolonged anesthetic effects when STX is combined with other drugs [28,29]. For instance, some liposomal formulations of STX (either alone or conjugated) were tested, and able to provide extended sciatic nerve block within rats, along with marginal systemic and local toxicities [28]. Apart from their application as therapeutic agents, STXs and their analogues may behave as markers to locate sodium channels, and constitute a research tool in the study of those channels. This could be an asset for sodium channel related-diseases, including diagnosis and treatment of patients suffering from those disorders.

Additionally, STXs have been reported as possessing antimicrobial activity (namely antibacterial, antifungal, antialgal and antiprotozoal). However, most such studies have resorted only to in vitro assays [30].

Some important analogues of STXs, e.g., gonyautoxins (GTXs), are produced by *Amphydinium* dinoflagellates, and exhibit a similar mode of action. They are paralytic toxins as well, and bind to VGSCs thus blocking the synaptic function. However, those biotoxins have proven a safe therapeutic approach against acute or chronic anal fissures. GTXs aid in sphincter relaxation, and thus function as pain killer [31]. GTX2 and GTX3 have also been used to treat chronic tension-type headache [32].

### 2.2. Tetrodotoxin

**Figure 2:**
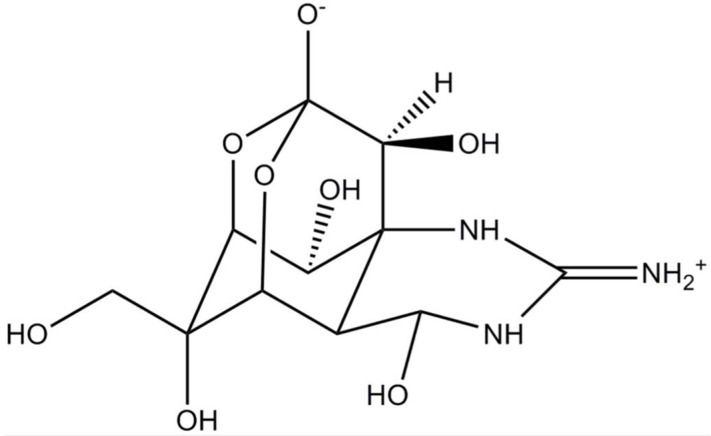
Chemical structure of tetrodotoxin (TTX) (adapted from [39]).

Tetrodotoxin (TTX) is traditionally known as the chief toxin in pufferfish, even though it is also produced by other marine animals (e.g., octopuses, gobies, sea stars) [17]. TTX-bacteria producing species were also identified in *Actinomyces*, *Aeromonas*, *Alteromonas*, *Bacillus* and *Pseudomonas* genera [33]; the only reference to a microalga producer is (dinoflagellate) *A. tamarense* [34]. There are at least 30 structural analogues of TTX reported to date, and their toxicity degree can differ according to the chemical structure [35]. TTX possesses a highly unusual chemical structure, containing a positively charged guanidinium moiety attached to a highly oxygenated carbon backbone. The carbon backbone of TTX consists of a 2,4-dioxyadamantane structure, decorated with 5 hydroxyl groups (Figure 2) [36]. This potent neurotoxin is of particular interest owing to its resemblance to saxitoxins (and analogues) in terms of effects. In fact, TTX has high affinity to VGSCs, thus blocking the propagating nerve and muscle action potentials [33]. Although TTX is extremely sensitive to Na_v_1.1, Na_v_1.2, Na_v_1.3 and Na_v_1.7, it can bind to other VGSCs subtypes to a lesser extent [37,38]. 

Besides VGSC key role in pain, TTX-sensitive subtypes have been implicated in normal and pathological pain [39]; TTX is indeed a powerful and selective drug, with an analgesic/anesthetic effect associated to its sodium channel-blocking properties. Several studies have demonstrated its effectiveness in many types of pain management protocols [40,41,42]. For instance, a powerful drug from this potent neurotoxin—Tectin^®^ by Wex Pharmaceuticals in Canada (http://www.wexpharma.com), is currently undergoing phase III clinical trials, with great success as pain controller in cancer patients [43]. Phase II clinical trials are also ongoing, aimed at assessing the efficacy of TTX against neuropathic pain produced by chemotherapy-induced peripheral neuropathy. In addition, a formulation—Tocudin^TM^, is under investigation for local and topical anesthesia, and preclinical testing will start soon [39]. The aforementioned TTX is currently obtained from pufferfish, since the production directly by dinoflagellates has proven unfeasible to date [3]. Additionally, TTX had been applied as moderator for acute heroin withdrawal symptoms (headache), with minor side effects [44]. 

### 2.3. Okadaic Acid and Dinophysistoxin

**Figure 3:**
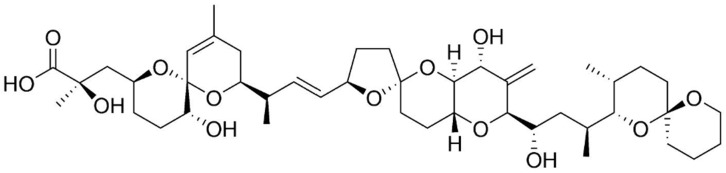
General structure of okadaic acid (adapted from [65]).

Okadaic acid (OA) and its derivatives, including dinophysistoxins (DTX)-1, 2 and 3, are polyether marine biotoxins found in various species of shellfish, and produced by several dinoflagellates [45]. They were first isolated from benthic dinoflagellates of *Prorocentrum* genus (e.g., *P. lima*, *P. concavum*, *P. belizeanum*, *P. maculosum*) and *Dinophysis* genus (e.g., *D. acuta*, *D. acuminate*, *D. fortii*) [46]; said biotoxins are potent protein phosphatase inhibitors, specifically serine and threonine phosphatases [10]. They are organized into long chain compounds (Figure 3), containing transfused or spiro-linked cyclic polyether rings with hydroxyl and carboxyl functions and methyl groups differing in number or position [16,47]. 

OA and DTXs are highly selective inhibitors of protein phosphatase types 1 (PP1) and 2A (PP2A); these enzymes have been implicated in a wide spectrum of reaction cascades. In fact, they were associated with metabolism, gene expression, cell proliferation, morphogenesis, ion regulation, neurotransmission, membrane transport, and cell cycle progression or secretion [48]. Blocking protein phosphatase activity results in hyperphosphorylation of many cell proteins, which in turn leads to dramatic effects upon normal regulatory pathways [49]. Therefore, OA and its analogues are extremely useful research tools for investigating cellular regulation processes, especially those related to reversible phosphorylation of proteins—such as signal transduction, cell division and memory [50]. 

Several studies, either in vitro or in vivo, have demonstrated the value of OA in medical/pharmacological research [51]. OA is a potent promoter of tumorgenesis [52], apart from having cytotoxic effects (apoptosis and cell growth inhibition) in many cell types—including intestinal cells, blood cells, neuronal cells, lung cells and hepatic cells. Its cytotoxic effects extend to embryonic development, immune and nervous system [51]. Due to inhibition of protein PP2A, OA has been used as an emerging tool for research on Alzheimer’s and other neurodegenerative disorders associated to memory-impairment [53,54,55,56,57]. Studies on diabetes, AIDS and cancer have also resorted to OA as biotoxin-model to elucidate several mechanisms associated thereto [58,59,60]. Furthermore, OA seems to have immunoregulatory potential, since it induces down-regulation of T-cell receptor expression—thus compromising T-cell responsiveness, and consequently immune response [61]. It has also the ability to stimulate inflammatory response via a considerable increase of interleukin 8 (IL-8) in HL-60 human cells [62]. Being a powerful tumor promoter, OA has also been claimed as angiogenic inducer in human endothelial cells, via the increasing activity of hypoxia-inducible factor-1 (HIF-1)—closely related to vascular endothelial growth factor [63].

Finally, OA from Prorocentrum has been shown to possess fungicidal activity—namely ability to inhibit growth of *Candida albicans* [64].

### 2.4. Yessotoxin

**Figure 4:**
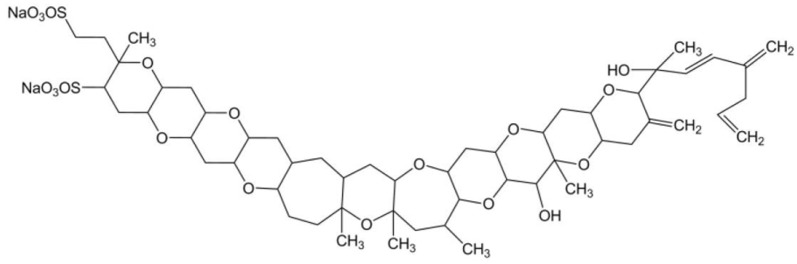
Chemical structure of yessotoxin (adapted from [89]).

Yessotoxin (YTX) is a marine sulphated polyether, produced by *Protoceratium reticulatum*, *Lingulodinium polyedra* [66] and *Gonyaulax spinifera* [67] dinoflagellate species. Data have been generated pertaining to more than 90 natural analogues of YTX recovered from cultures of *P. reticulatum*; the chemical structure of this toxin has already been elucidated, yet the structures of most of its analogues remain to be resolved [68]. This toxin is composed by a distinctive ladder-shape formed by several ether rings of different sizes, and a terminal acyclic unsaturated side chain consisting of 9 carbons and 2 sulfate ethers (Figure 4). The core structure is liposoluble, but the two sulfate groups convey amphoteric features to the molecule. Owing to this characteristic, such a compound is considered one of the most polar among the otherwise lipophilic toxins [69,70].

YTX and its analogues are particularly interesting tools for probing biological and pharmacological mechanisms [71]; they indeed can interfere with several biological apoptotic pathways in a variety of cellular systems, including tumor cells [72]. YTX can also induce non-apoptotic cell death in BC3H1 myoblast cells, primary cortical neurons and glioma cells [71,73]. Some studies have pointed at YTX as a potent phospodiesterase (PDE) activator [74,75], although the exact mode of action remains uncertain [17]. PDEs play a key role as regulators of signal transduction, mediated by such second messenger molecules as cyclic adenosine monophosphate (cAMP) [76]. Moderate modulation of intracellular calcium and cAMP levels [75,77], promotion of caspase protein activation [78], permeability transition through mitochondria [79], alteration of cytoskeleton (viz. selective disruption of F-actin microfilaments) [80,81], and fragmentation of adhesion proteins (specifically E-cadherin) [82], are among the reported YTX effects—dependent on cell line used and treatment duration [83]. Recently, YTX was found to induce mitotic catastrophe and genetic alterations—which may be of interest for control of tumor progression [84]. Additionally, Tobío et al. [85] have claimed regression of melanoma tumor cells in mouse cells in vivo, along with negligible toxicity. YTX may also pay a minor role as anti-allergenic and -asthmatic drug, even though the mechanism underlying these therapies remains poorly understood [85]. YTX seems to interfere with the immune function, since it reduces phagocytic activity on J774 cell line and increases expression of cytokines in J774 phagocyte mammalian cells [80]. Moreover, it appears to regulate the immune-effect on T-lymphocyte EL-4 cells via reversible down-regulation of the T-cell receptor complex [49,61]. Regarding other pharmacological effects, YTX and its analogues may be employed as therapy for Alzheimer’s disease [73]. These compounds have improved the levels of t- and β-amyloid—both insoluble structures that appear in the brain and are responsible for triggering said disease [86]. Furthermore, YTX may aid in treatment/prevention of lipid and glucose metabolism-associated diseases [73]. Early studies unfolded fatty degeneration, with alterations in pancreas and liver [87]; significant transcriptional alterations in lipid and glucose metabolism were in fact described in glioma cells [88].

A markedly increased activity against fungi and yeasts was reported when YTX had been chemically desulfated, with reduced toxicity toward mouse species. Therefore, expectations remain high with regard to YTX produced by dinoflagellates as promising candidates for novel and potent antifungals [64]. 

### 2.5. Pectenotoxin

**Figure 5:**
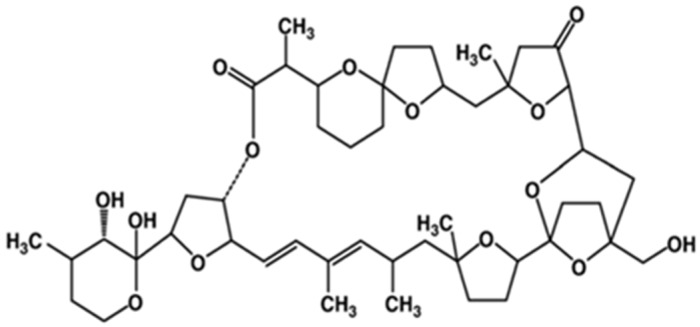
General structure of pectenotoxin (adapted from [97]).

Pectenotoxin (PTX), together with its analogues, are polyether macrolide compounds produced exclusively by *Dinophysis* species (e.g., *D. fortii*, *D. acuta*, *D. tripus*, *D. acuminate*, *D. caudate*, *D. rotundata*, *D. norvegica*) [65]. More than 20 analogues have been isolated to date [90], with disparate toxicological potency. Their common structural features include a spiroketal group, three oxolanes, a bicyclic ketal and a six-membered cyclic hemiketal (Figure 5) [91].

In general, they exhibit strong toxicity against hepatocytes; their action mechanism in vitro and in vivo encompasses actin filament depolymerization, leading to notorious effects upon cytoskeleton arrangement [92,93]. As a result, PTX causes cell cycle arrest and apoptosis [94]—being particularly effective against tumor cells, rather than normal cells of the same tissue [92]. For instance, PTX-2 has demonstrated antitumor activity against human lung, colon and breast cancer cells [95]; and was claimed as potent chemotherapeutic agent against p53-deficient tumors [96].

### 2.6. Ciguatoxin

**Figure 6:**
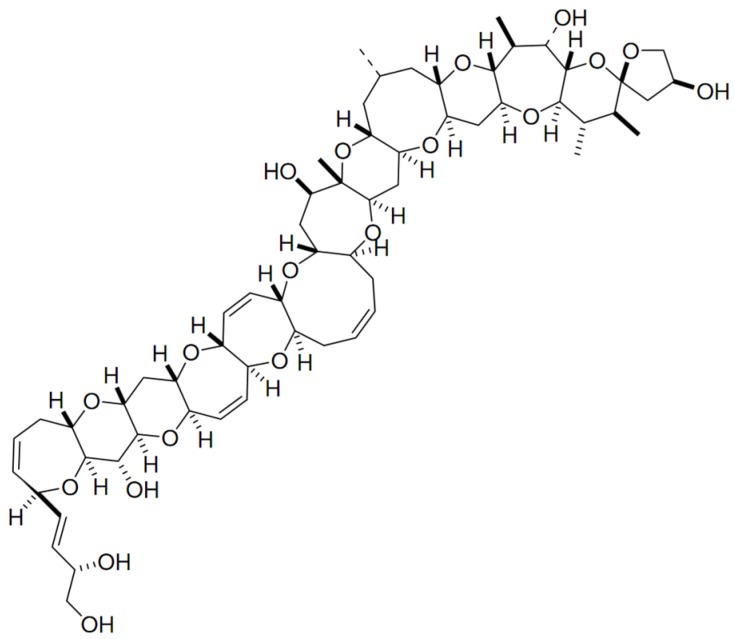
General structure of ciguatoxin (adapted from [97]).

Ciguatoxin (CTX) belongs to the group of marine polycyclic ether biotoxines implicated in ciguatera fish poisoning outbreaks. This fat-soluble substance is produced by certain strains of benthic *Gambierdiscus toxicus*, and may arise in fish from a biotransformation of gambiertoxins (e.g., maitotoxins) as precursors [98]. It accumulates throughout the food chain up to higher predators, and may ultimately reach human consumers—thus causing neurological, gastrointestinal and cardiovascular disorders [99,100,101,102]. More than 20 analogues have been found in the Pacific area, and multiple forms of CTX with minor molecular differences and toxicities were found in Caribbean waters [100] and the Indian Ocean [103]. This group of lipid-soluble polyethers are composed by a distinct and long semi-rigid ladder-like structure comprising several trans/syn ether rings at different sizes (Figure 6) [104,105]. 

CTX is a potent modulator of site 5 on VGSCs of a wide variety of cells, following a mechanism similar to BTX; however, it is one hundred-fold more potent than BTX in eliciting repetitive neuron firing [105,106,107]. This compound is able to shift the potential activation (hyperpolarization), and change gating properties by activating VGSCs in a persistent way (from nM- to pM-concentration range), thus resulting in an enhanced Na^+^ inward current directly into excitable cells accompanied by an efflux of K^+^ [108]. The plasma membrane is unable to maintain the internal conditions leading to modification of bioenergetics mechanisms, bleb formation and cell and mitochondrial swelling [100,109]. They were found to significantly slow nerve conduction rate, and reduce amplitude in human nerves—consistent with abnormal and extended Na^+^ channel opening in nerve membranes in vivo [110,111]. In addition, some normal cellular mechanisms counteract this effect when Na^+^ ions move into the cytosol—while evoking Ca^2+^ capture and increase of its level inside the cells. This calcium acts as a second messenger, thus disrupting important ion-exchange systems. Hence, elevated muscle contraction, especially in cardiac tissue, and high fluid secretion by gastrointestinal cells are observed [100]. 

In neuromuscular junctions—and apart from elicitation of repetitive action potentials, said biotoxin may cause a dramatic increase in asynchronous acetylcholine release, and impair synaptic vesicles [112]. Other effects of CTX encompass catecholamine secretion from neuroendocrine cells [113]. The diversity of human symptoms associated to ciguatera may arise from the different affinity of CTX for the various VGSCs (Na_v_) subtypes. The fact that CTX is reported as discriminatory of several N_av_ channel subtypes—particularly Na_v_ 1.2 and 1.3 (brain), Na_v_ 1.4 (skeletal muscle), Na_v_ 1.5 (heart), Na_v_ 1.6 (motor neuron, smooth muscle), Na_v_ 1.7 (peripheral nervous system) and Na_v_ 1.8 (peripheral nervous system) [114,115,116], make this biotoxin a resourceful tool to investigate the biological function and structure of said ion channels in further depth [107]. Note that these types of channels underlie several human diseases and channelopathies (e.g., chronic pain, cardiac arrhythmias, epilepsy, and even cancer) [116,117]. 

### 2.7. Maitotoxin

**Figure 7:**
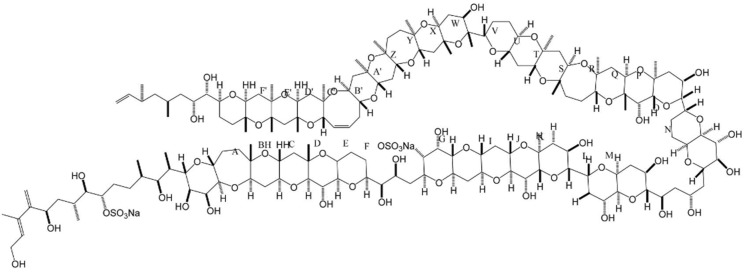
Chemical structure of maitotoxin (adapted from [9]).

A unique polyketide-derived polycyclic compound, maitotoxin (MTX), has been recognized for its potential to aid in research in chemistry and biology [118]. It has indeed been reported as the largest and most potent secondary metabolite ever isolated; its acute toxicity against mice far exceeds that of tetrodotoxin [118,119]. *Gambierdiscus* (i.e., *G. toxicus*, *G. australes*, *G. pacificus*) is the only genus found so far that produces the three existing forms of MTX (i.e., MTX-1, -2 and -3) [120,121,122]. Recently, a new analogue—MTX-4, was found that is by *Gambierdiscus excentricus* (from Canary Islands) [123]. In addition, some MTX precursors are produced by *Amphidinium carterae*, *Prorocentrum* sp., *Ostreopsis* sp., *Thecadinium* sp. and *Coolia monotis*. MTX is water-soluble, and entais a complex ladder-shaped polycyclic molecule composed by several hydroxyl and sulphate groups (Figure 7) [9,119]. This toxin is believed to cause ciguatera, but with symptoms different from those caused by ciguatoxins—due to an apparently distinct mode of action [124]. In early reports, direct involvement with calcium voltage gated-channels was claimed for MTX. Nonetheless, other observations [125] indicate that MTX binds to the cell membrane (lipophilic domain), thus inducing non-selective influx of ions into the cells—which, in turn, activates the voltage-sensitive calcium channels [125]. Unfortunately, the specific target of this compound remains unknown [118]. MTX is believed to be a powerful disruptor of Ca^2+^ homeostasis, with a multiplicity of pharmacological effects upon several cell lines [119]. Its ability to trigger intracellular cascades of events—e.g., membrane depolarization in excitable cells [126], insulin [127] and neurotransmitter secretion [128,129], phosphounisitide breakdown (important in cell lipids and cell signaling) [130], programmed cell death [131], and fertilization [132,133], justify why this compound is a powerful tool for research in cell biology, namely when attempting to elucidate Ca^2+^-dependent cellular processes [119]. MTX has also been suggested to play a role in innate immune responses and inflammation in vivo [119,134]. Its toxic effect seems to trigger a mediated inflammation response via secretion of pro-inflammatory cytokines IL-1β; this may be viewed as an interesting tool for studying specific components of innate immune response and/or the physiology of inflammatory effector cells [119]. More recently, MTX was claimed as selective activator of a specific transient receptor potential (TRP) in *Xenopus laevis Oocytes*; TRP channels are apparently involved in the regulation of non-selective cation channels. MTX may be of potential use for further studies in these type of biological channels [135]. 

### 2.8. Palytoxin and Ostreocin

**Figure 8:**
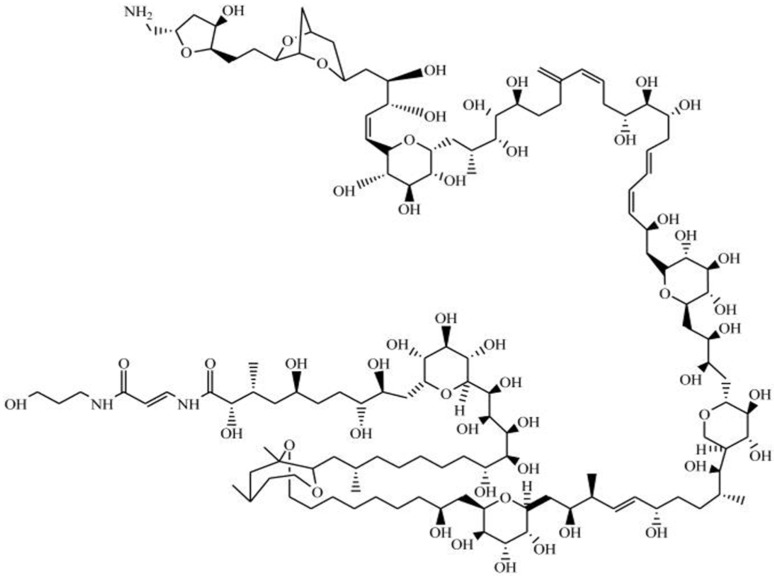
Chemical structure of palytoxin (adapted from [139]).

Palytoxin (PLTX) is a large and complex polyether compound, with a remarkable biological activity [9]—including a wide spectrum of pharmacological effects [136]. Originally isolated from the zoantharians of *Palythoa* genus [137], PLTX is also found in a number of marine organisms, including all species of *Ostreopsis* dinoflagellates (e.g., *O. siamensis*, *O. mascarenensis*, *O. lenticularis*, *O. ovata*, *O. fattorussoi*) [138]. This complex polyhydroxylated marine-derived molecule has both lipophilic and hydrophilic regions, and is composed by the longest continuous carbon atom chain of any known natural product (next to maitotoxin). Along its backbone, it possesses several hydroxyl groups, two diene motifs, and two hydrophobic hydrocarbon chains, among other structural features (Figure 8) [137,139].

PLTX-like compounds produced by dinoflagellates are commonly known as ostreocins. They are quite toxic against mammals; PLTX and its analogues may actually be the most lethal marine toxins known at present [136]. These compounds affect cellular function via inhibition of ATPase Na^+^/K^+^ pump—a transmembrane enzyme, essential to maintain ion homeostasis in excitable and non-excitable tissues [140]. PLTX accordingly restrains active transport of ions, and blocks the electrochemical gradient generated across the cell membrane—thus transforming the pump into a non-specific, permanently open ion channel. This leads to membrane depolarization and massive influx of calcium into the cytosol, thus compromising several cellular functions [139]. A number of studies have also indicated a wide variety of secondary pharmacological actions—including hemolysis, modulation of some neurotransmitters (norepinephrine and/or acetylcholine), and activation of pro-inflammatory signaling cascades (i.e., release of histamine and prostaglandin-E2) [141]. PLTX and ostreocin-D are apparently also involved in actin cytoskeleton distortion and dynamics, as proven via different cellular models (e.g., intestinal and neuroblastoma cells) [139,142]. The data so far available suggest that PLTX and ostreocin-D can modulate the unassembled actin pool, by activating signal transduction pathways not related to Ca^2+^ influx [143]. Despite these two compounds sharing the same molecular target, a few small structural differences can significantly reduce cytotoxicity and hemolytic potency in the case of ostreocin-D [144].

PLTX has also been claimed as powerful tumor promoter, and accounts for several effects, e.g., stimulation of arachidonic acid metabolism, modulation of epidermal growth factor (EGF) receptor, production of prostaglandins, and activation of mitogen-activated protein (MAP) kinase cascades [140]. In addition, patent EP3087172 claims that a pharmaceutical formulation with PLTX (sourced from *Palythoa clavata* polyps, comprising *Symbiodinium* dinoflagellate) is suitable for therapeutic use against lymphoblastic or myelogenous leukemia (Table 1).

Discovery of novel properties of PLTX and PLTX like-compounds, from marine dinoflagellates, may constitute a potential pathway for biotechnological characterization of living systems (e.g., focused on pump mechanism) [145,146]; and it may set the basis for a promising form of anti-tumor therapeutics.

### 2.9. Gambierol

**Figure 9:**
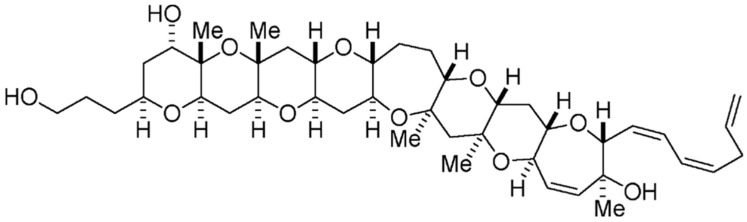
Chemical structure of gambierol (adapted from [162]).

Produced by *Gambierdiscus toxicus* dinoflagellate, gambierol is part of the group of polycyclic ethers; it is believed to be one of the components involved in ciguatera fish poisoning [147]. Its chemical structure resembles those of cigatoxins and brevetoxins, with potent neurotoxicity [105,148]; and is characterized by eight ether rings with two pyranyl rings—with methyl groups in a 1,3-diaxial orientation (Figure 9) [149,150]. As happens with other marine polyether metabolites, its scarcity from natural sources has hampered further biological studies. Chemical synthesis has been attempted to overcome these difficulties, and assure a higher availability of this substance for tests in vitro and in vivo [151]. Unlike such other marine polycyclic toxins as ciaguatoxins, gambierol does not envisage VGSCs as main targets [152]; it instead exerts a powerful modulatory action upon voltage-gated K^+^ channels (K_v_) [153,154]. It acts as an intermembrane anchor, by binding specifically to K_v_3.1 channels that, in turn, block K_v_ channels. As a consequence, the channels remain closed, thus lowering K^+^ ion currents [155]. Furthermore, it is able to evoke cytosolic calcium oscillations in cerebrocortical neurons, as an outcome of channel K_v_ inhibition [156,157,158]. Cao and co-workers [156] demonstrated that gambierol also induces outgrowth of neurites in a bidirectional manner; this may be promising for victims of neural injury.

Despite its toxicity, this compound and its (less toxic) synthetic analogues have been suggested as new drugs for immunotherapy [159,160]. In fact, K_v_3.1 channels play a key role on modulation of Ca^2+^ signaling, which in turn induces T-cell proliferation, immune activation and cytokine production. Said channels are believed to be therapeutic targets of T-cell mediated autoimmune diseases [159,161]. Given its particular capacity to block K_v_3.1 channels, gambierol is an interesting compound for application as immunosupressor in dysfunctional immune system diseases, such as multiple sclerosis, diabetes mellitus type 1 and rheumatoid arthritis [159,161]. Gambierol and two of its analogues (tetra and heptacyclic forms) are promising molecules for modulation of Alzheimer’s disease hallmarks in primary cortical neurons. It was shown that β-amyloid and/or tau hyperphosphorylation overexpression can be reduced by gambierol, both in vitro and in vivo (Table 1).

### 2.10. Brevetoxin

**Figure 10:**
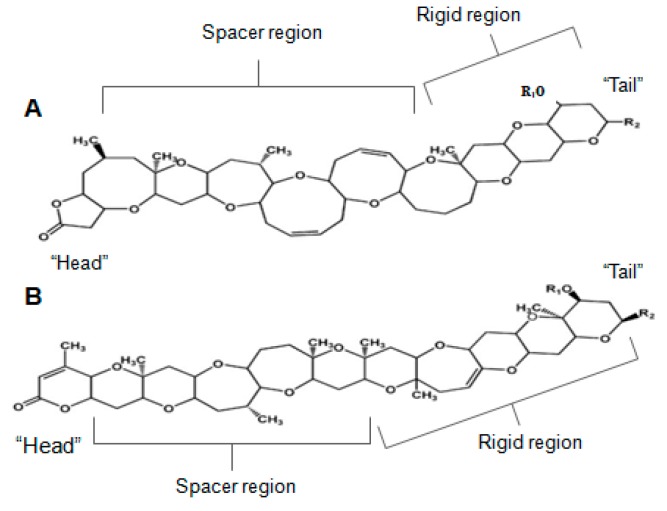
Chemical structures of (**A**) brevetoxin type-A and (**B**) brevetoxin type-B (adapted from [185]).

Brevetoxin (BTX) is a ladder-like polycyclic ether, recognized for its powerful neurotoxic and ichthyotoxic features [9]. BTX originates from unarmoured dinoflagellate *Karenia brevis* (formerly known as *Gymnodinium breve* or *Ptychodiscus brevis*) [163]. BTX has nine analogues, classified based on its backbone structure: A-type or B-type [47,164]. More recently, a few other analogues were found in fish-killing species that belong to the class of raphidophytes [165,166,167]. The brevetoxin type-A is a decacyclic molecule, consisting in 10 transfused rings; and breveotxin type-B is an undecacyclic molecule, with 11 transfused rings—both with a functional lactone in one of the terminal rings, denominated “head”. They have also a strictly rigid region in the terminal four rings, a spacer region that separates the rigid region from the A-ring lactone, and a side chain allowing modest modification at the molecule terminus, or “tail” (Figure 10) [168]. Alterations in any type of such regions may induce modification in their activity, or induce significant loss in binding activities [169]. BTX binds to the α-unit of VGSCs, specifically site 5 [107,170,171,172].

Several BTX analogues and derivatives possess distinct toxicity efficacies, depending on binding affinity to VGSCs on site 5 [168,173]. Instead of blocking the channel, BTX action produces persistent activation of VGSCs, and their extended opening leads to prolonged Na^+^ entry into the cells [174,175,176]. This causes membrane depolarization at the resting membrane potentials—which triggers repetitive firing or excitatory cellular responses, and leads to such other physiological disturbances as Ca^2+^ influx [116,173,177,178]. Mattei and co-workers [109] also identified water movement across the membranes in myelinated nerve fibers; however, the regulatory mechanisms involved could not be elucidated. Another effect is enhanced release of neurotransmitters from autonomic nerve endings, acetylcholine in particular—which lead to smooth tracheal contraction [179]. In fact, during exposure to this substance, such symptoms as respiratory irritation (cough, nose irritability, congestion), bronchoconstriction and/or asthma attacks were observed in healthy individuals, but were more serious in airway-disease sensitive persons [180]. Therefore, it appears to yield an immune response, and play a major role in allergic inflammation in pulmonary tissue [181,182]. Sas and Baatz [182] suggested a primary inflammatory response in alveolar macrophage cells, mediated by the increase of cytokines (such as TNF- and IL-2) involved in immune cell activation and phagocytosis promotion. Another study [181], involving mouse bone marrow-derived mast cells, has shown that BTX can directly activate mouse mast cells—thus leading to degranulation, as well as inflammatory cytokine production involving Ca^2+^ signaling. Conversely, other authors claimed that exposure to BTX impairs the immune function, thus leading to reduced phagocytosis activity, decreased plaque-forming ability and/or decreased lymphocyte proliferation [183]. This biotoxin has also been described [178,183] to affect cell proliferation in a dose-dependent manner, cause cell death through an apoptotic mechanism, and possess genotoxic features. 

Based on its neuro-activation properties, BTX-2 has been found to behave as neuronal stimulator, able to increase neuronal plasticity. It might thus support advances in pharmacological treatment aimed at recovering brain function after stroke or other traumatic brain injury [184]. Furthermore, a therapeutic formulation based on BTX derivatives has been designed to regulate such diseases as cystic fibrosis and mucociliary dysfunction related to increased mucus transport (Table 1).

### 2.11. Azaspiracid

**Figure 11:**
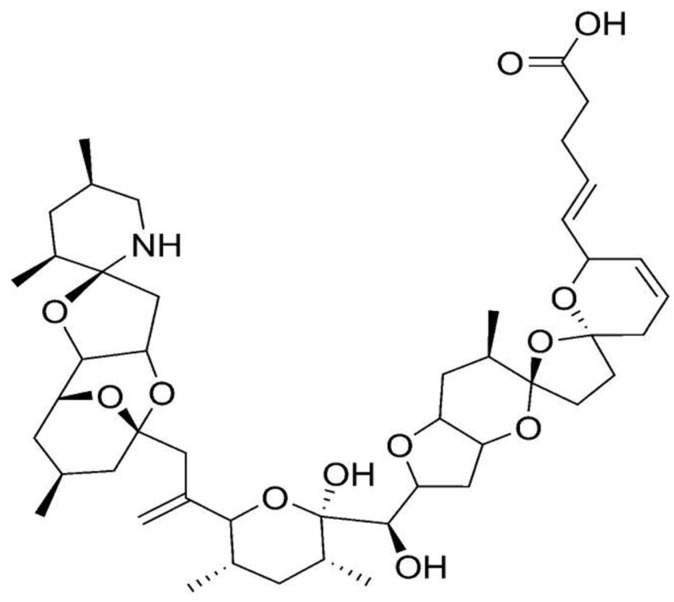
Chemical structure of azaspiracid-1 (AZA1) (adapted from [65]).

Azaspiracid (AZA) is a recently discovered polyether phycotoxin, quite toxic for mammal systems [10]. AZA is responsible for azaspiracid poisoning, and is produced by dinoflagellate *Azadinium* genus (e.g., *A. spinosum*, *A. poporum*, *A. dexteroporum*) [186,187,188]. Among its increasing number of derivatives (over 30 so far) [189], Azaspiracid-1 (AZA1)—the first compound to be isolated and the one with major toxicity in humans, appears to be the most important, followed by AZA2 and AZA3 [190]. AZA toxin consists in a highly hydroxylated linear carbon chain with a tri-spiro ring assembly, together with a unique cyclic amine (or aza group) and a carboxylix acid group (Figure 11) [188,191]. The unique cyclic amine is a structural feature that differentiates AZA from the other dinoflagellate toxins [9]. Its mechanism of development of toxic effects is not fully understood [17], yet AZA is expected to possess a strong biotechnological significance. Toxicological studies in vivo and in vitro have unfolded several aspects of cell biology that can be affected thereby [192]. AZA presents indeed cytotoxicity against several human cell types [193], as well as teratogenicity to finfish [194]. It has also the ability to induce alterations on cell morphology and cytoskeleton structure, particularly on the E-cadherin system [65,192]. Furthermore, it was reported to be a potent activator of c-Jun-*N*-terminal kinase (JNK) and caspases—implicated in stress-signaling pathways, such as cell damage, apoptosis, and cytoskeleton regulation; and as an effective modulator of intracellular cAMP and calcium levels [195,196,197]. Other known effects relate to altered gene expression patterns in cells, and inhibition of cell cholesterol levels (particularly in T-lymphocyte cells) [198,199]. Finally, AZA apparently affects potassium ion channels [200].

### 2.12. Gymnocin

**Figure 12:**
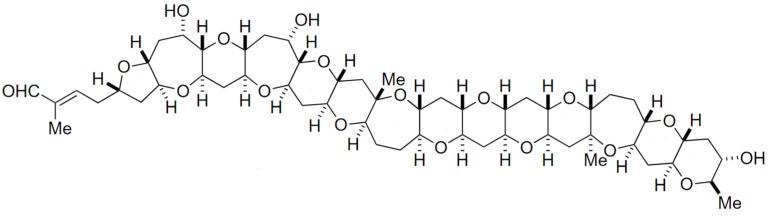
Chemical structure of gymnocin-A (GYMA) (adapted from [202]).

Gymnocin-A (GYMA) is a rare and complex polyether toxin, isolated from the red tide dinoflagellate *Gymnodinium mikimotoi*. This toxin is composed by fourteen contiguous polyether rings, with 2-methyl-2-butenal side-chains (Figure 12) [201]. It is weakly toxic upon fish, but quite cytotoxic against P388 mouse leukemia cells. Several other forms of GYMA have meanwhile been isolated, including Gymnocin-B bearing even higher cytotoxicity [202].

### 2.13. Karlotoxin

**Figure 13:**
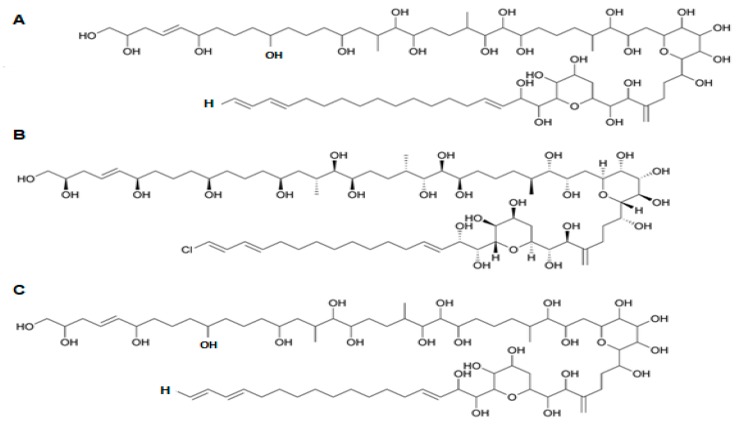
Chemical structure of (**A**) kartoxin-1 (KmTx-1); (**B**) karlotoxin-2 (KmTx-2) and (**C**) karlotoxin-3 (KmTx-3) (adapted from [218]).

Karlotoxin (KmTx) is a linear polyketide toxin [203], synthesized exclusively by *Karlodinium* genus; *K. veneficum* sp. is indeed considered as the main source of this biotoxin [204]. Different strains of this dinoflagellate, collected across distinct geographic locations produce several forms of KmTx with differing physicochemical properties [205,206]. Its chemical structure has recently been elucidated, and three groups of KmTxs accordingly emerged—differing mostly in length of lipophilic arm, a structural feature that apparently modulates haemolytic activity [207,208,209,210,211]. In KmTx 1, the side chain has 18 carbons in length, whereas KmTx 2 is two carbons shorter and KmTx 3 differs from KmTx 1 in having one less methylene group in the saturated portion of its lipophilic arm (Figure 13) [210,211]. Surprisingly, KmTxs have remarkable structural similarities with amphidinols—bioactives produced by dinoflagellate of genus *Amphidinium* sp. [212], characterized by long carbon chains with multiple hydroxyl groups and polyolefins. KmTx chemical structure consists of a complex hairpin-like structure, with three distinct regions: a polyol arm bearing variable hydroxylation and methylation; a hinge region containing two pyran rings; and a lipophilic arm with a terminal diene [213].

KmTx holds a range of activities, such as haemolytic, cytotoxic, ichthyotoxic and antifungal [206,207,214,215]. Its similarity to amphidinols (produced by *Amphidnium* genus) suggests a similar mode of action, based on membrane cell permeabilization [203,206,216]. KmTx acts on cell membranes via pore formation, which disrupts cell osmotic balance and eventually leads to cell death. A study on Km-Tx2 revealed that lysis is preceded by permeabilization of the plasma membrane to various cations, including Ca^2+^, K^+^ and Na^+^ [207,215]. The bioactivity of these compounds is dependent on the sterol composition of the target cell [204,205]. During pore formation, KmTxs selectively bind to 4-desmethyl sterols (e.g., cholesterol or ergosterol), whereas cells containing 4α-methyl sterols (e.g., gymnodinosterol and brevesterol) are immune to said biotoxin. This might explain why KmTx is capable of lysing animal cells, fungi and/or protists, but leaves *K. veneficum* cell membranes intact; dominance of 4α-methyl sterols in the latter may be the key to this lack of autotoxicity [205,217]. The KmTx properties to trigger formation of pores in cholesterol-containing cell membranes convey a noteworthy potential to treat several human health conditions, including coronary heart disease (CHD). Furthermore, KmTx may be formulated as a new chemotherapeutic agent for cancer control. Cholesterol acts as both adhesive and spacer unit between the sphingolipids that hold the lipid raft in cell membranes together—being critical for biological competence. In some solid tumor lines, e.g., breast and prostate cancer cells (two of the most widespread cancer forms worldwide), much more lipid rafts are present than in their healthy counterparts—so they are more sensitive to cholesterol depletion-induced cell death [204,207].

### 2.14. Cyclic Imine Toxins (Spirolide and Gymonodimine)

**Figure 14:**
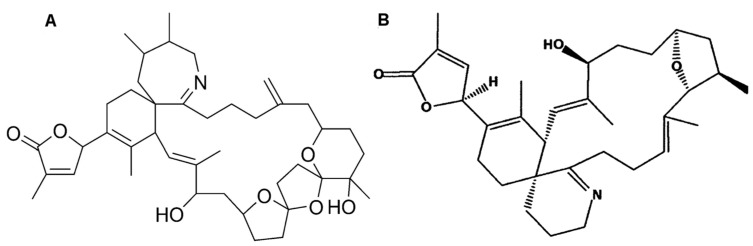
Chemical structure of (**A**) 13-desmethyl spirolide C (adapted from [65]) and (**B**) gymnodimines A (GYMA) (adapted from [235]).

Spirolide (SPX) and gymnodimine (GYM) are biotoxins belonging to the cyclic imine group, known to be “fast action toxins”—i.e., able to produce potent and rapid death in rodents [10]. SPX is synthesized by *Alexandrium ostenfeldii/peruvianum* [219,220], and also by *Karenia selliformes* [221]; and 16 isoforms have been identified to date [222]. This toxin is a macrolide characterized by a cyclic imine group; the most common analogue found is 13-des-methyl-C-spirolide (see Figure 14A) [89]. The latter is included in the first of the four groups of SPXs, defined as per functional group present. For instance, the first group includes ten forms having a characteristic 6,5,5-spiroketal ring system (i.e., SPX-A, SPX-B, SPX-C, SPX-D, and 13-des-methyl-C-spirolide, among others). The second group of spirolides (SPX-E and -F) chemically resembles SPX-A and SPX-B, except for lack of toxicity; instead of the cyclic imine group, they have other structural component (acyclic aminoketone) [223,224,225]. The third and fourth groups (i.e., SPX-G or SPX-I) are represented by analogues resembling SPX-C and -D, except that some compounds have a 6,5- instead of the 6,5,5-spiroketal ring system [225,226]. Generally speaking, SPX toxins have confirmed their major activity upon muscarinic and nicotinic acetylcholine receptors, along with damage to astrocytes and neurons that negatively disturb the central nervous system [227]. 

GYM (including Gymnodimine-A, and its two analogues GYM-B and -C) [221] are produced by gymnodinoid dinoflagellates, specifically *Karenia selliformis* (formerly named *Gymnodinium selliforme*) [221,228]. A fourth analogue of GYM—12-methylgymnodimine—was isolated from *Alexandrium ostenfeldii* [229]; GYM-D was recently found as new analogue [230]. GYM molecules present typically a six-membered cyclic imine, with no methyl substituents in spiroimine ring system, and with such typical fragments as tetrahydrofuran ring and unsaturated lactone (Figure 14B) [230]. 

Both SPXs and GYMs contain a unique cyclic imine ring, hypothesized to be their pharmacophore moiety [223]; it might be responsible for activation of L-type calcium channels of brain receptors [228]. Nevertheless, recent studies have demonstrated that these compounds can target neuronal and muscular nicotinic acetylcholine receptors with high affinity [231,232]. Such dinoflagellate toxins are accordingly proposed as additional tools to elucidate structural domains on various acetylcholine receptors (AChR); and to advance understanding of interactions between antagonists, and nicotinic and muscular AChR [232]. SPX and GYM mechanisms of action appear to be similar, yet they remain largely undisclosed [10]. Some reports have shown that GYM (combined with OA) can be used therapeutically to enhance the anti-cancer effects of chemotherapeutic agents—many of which work partially via toxicity against tumor cells. It was demonstrated that GYM can sensitize cells to apoptotic stimuli Neuro2a neuroblastoma cell line [233]. GYM has also been claimed to cause a reduction of β-amyloid levels and tau phosphorylation, which could potentially contribute for treatment of degenerative diseases [234]. 

### 2.15. Gambieric Acid (Bioactive)

**Figure 15:**
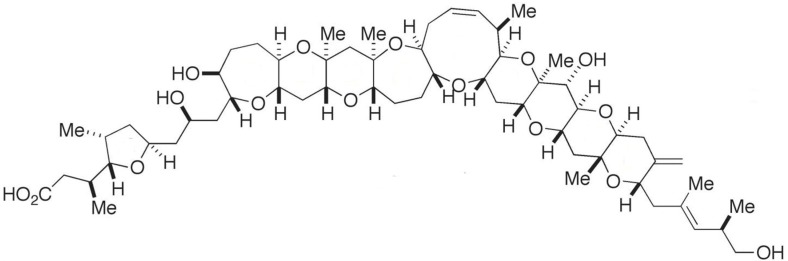
Chemical structure of gambieric acid (GA-A) (adapted from [148]).

Gambieric acid (GA) belongs to a family of marine polyether natural products, originated in dinoflagellate *Gambierdiscus toxicus*; gambieric acids A, B, C and D have been isolated from their culture broth [236]. Such compounds are composed by nine trans-fused ether rings of six, seven and nine members, and one isolated tetrahydrofuran ring (Figure 15) [237]. They are potent antifungal agents, displaying a remarkable activity against filamentous fungi, while being ineffective against bacteria or yeasts. A study involving GA-A and GA-B has confirmed their potency against fungus *Aspergillus niger*—more than 2000-fold that of amphotericin B, a common antifungal drug [238]. On the other hand, GA does not show substantial toxicity against cultured mammalian cells, or even in vivo. Mice subjected to doses of 1 mg per kg of body weight, via intraperitoneal injection, did not develop abnormal reactions or considerable toxicity—despite GA sharing structural features with polycyclic ethers, e.g., ciguatoxins, brevetoxins, gambierol and maitotoxin [239]. GA-A is able to displace binding of tritiated brevetoxin B to voltage-gated sodium channels in excitable membranes—even though its binding affinity is significantly lower than those of brevetoxins and ciguatoxins [240].

### 2.16. Goniodomin A (Bioactive)

**Figure 16:**
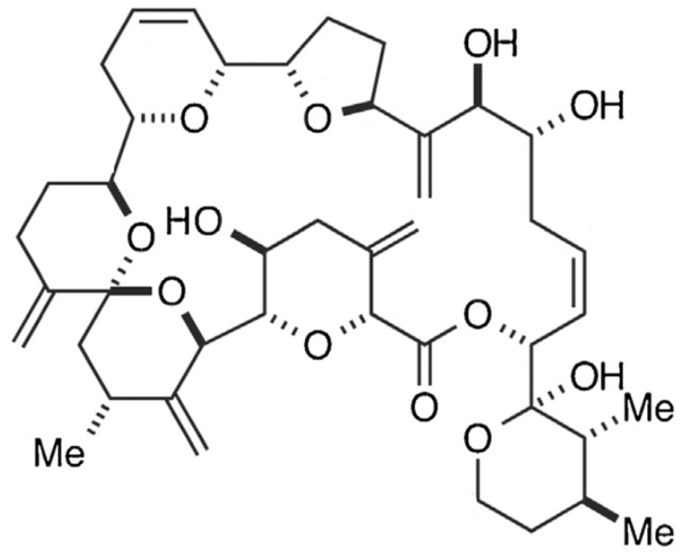
Chemical structure of goniodomin-A (GDA) (adapted from [247]).

Goniodomin A (GDA) is an antifungal polyether macrolide, produced by *Alexandrium* genus, namely *A. hiranoi* [241], *A. monilatum* [242] and *A. pseudogonyaulax* [243]. Pharmacological studies have indicated its strong effect upon cytoskeleton reorganization [242]. Structurally similar to pectenotoxin, this compounds is composed by a macrolide lactone ring, with a spirocetal ring and a hemiacetal ring attached thereto (Figure 16) [244]. This compound inhibits angiogenesis (regeneration of vessels) by inhibiting endothelial cell migration and basic fibroblast growth factor (bFGF)-induced tube formation—in part via inhibition of actin reorganization. Angiogenesis is also inhibited by GDA in vivo [245]. Mizuno et al. [246] reported that GDA affects the actin state in astrocytoma cells, causing cell morphological changes by increasing filamentous actin. GDA was shown to induce increase of filamentous actin content in Clone 9 rat hepatocytes, as well as cytotoxicity against human neuroblastoma cells. Goniodomin B, an analogue of GDA, seems to have effects similar to GDA but less potent [247].

GDA can be a useful tool for analysis of the relationship between structure and function of actin. On the other hand, this substance has shown antifungal activity against *Candida albicans* and *Mortierella ramannianus* [241].

### 2.17. Amphidinolide (Bioactive)

**Figure 17:**
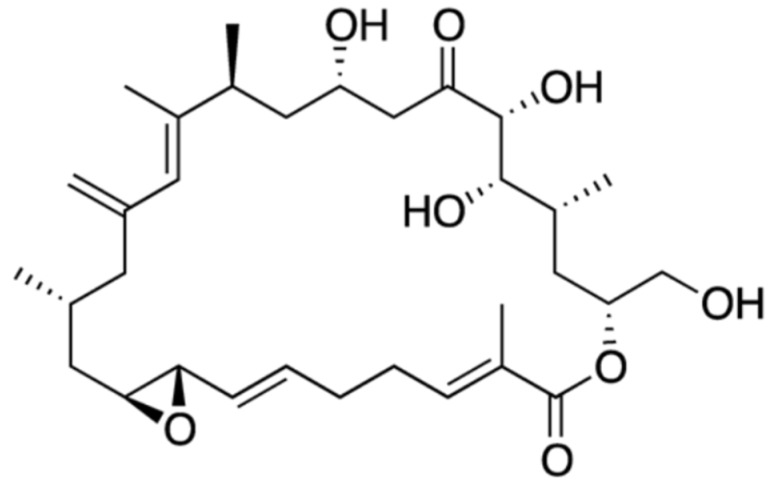
Example of chemical structure of amphidinolide-H (adapated from [249]).

Amphidinolide (AMP) constitutes a group of citotoxic macrolides, produced by symbiotic dinoflagellates of *Amphidinium* genus [248]; more than 40 AMPs were identified to date [249]. Such compounds exhibit great variation in size of macrocyclic lactone rings, from twelve- to twenty seven-membered systems (Figure 17) [250,251], with other unique structural features that make them quite complex molecules. In general, they exhibit potent cytotoxicity against murine lymphoma L1210, and human epidermoid carcinoma KB cells in vitro [248]. Among all AMPs, the noteworthy anti-tumor capacity of AMP-N and AMP-H is likely related to distinct inhibitory patterns. While AMP-N seems to have more affinity for mitochondria of malignant cells, AMP-H apparently targets actin cytoskeleton [252]. These compounds are expected to lead to new anticancer drugs—but again their limited availability has hampered more detailed studies [248,251]. Other related compound, caribenolide-I, was reported to possess strong cytotoxic activity against human colon tumor cell line and murine tumor P388 in vivo [97].

### 2.18. Amphidinol (Bioactive)

**Figure 18:**
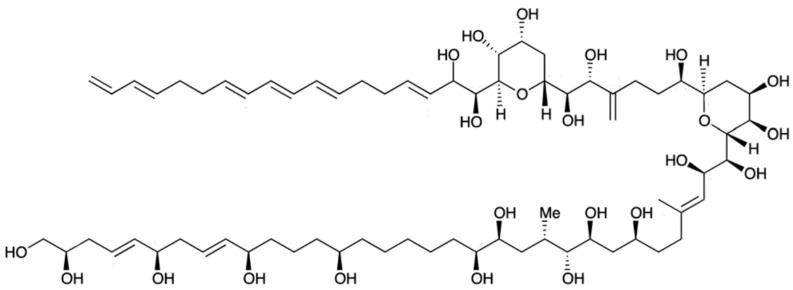
Chemical strucuture of Amphidinol-3 (AM3) (adapted from [264]).

Amphidinol (AM) belongs to a unique group of polyhydroxy polyene compounds, produced by *Amphidinium* species (e.g., *A. klebsii*, *A. carterae*); it possesses antifungal and haemolytic properties [253]. At least 23 AMs (including 7 analogues) [254,255,256] have been reported, ever since amphidinol 1 (AM1) was first isolated from *A. klebsii* in 1991 [257]. AMs belong to a large family of long-chain linear polyethers, presenting noteworthy potent biological activities—including antifungal, cytotoxic, and haemolytic properties [255]. Amphidinol-3 (AM3) (Figure 18)—the first of this series of compounds to be fully elucidated [212,258], notably exceeds other derivatives in terms of both activities. It was initially believed that AM permeabilizes phospholipid membranes, by interacting directly with such bilipidic layer and forming ion-permeable pores across the membrane (toroidal pore model) [203,259]. A recent study revealed, however, that AM3 is able to perforate membranes by specific molecular recognition, but without apparent disruption of the membrane itself. A novel action mechanism based on a barrel-stave pore model has been proposed for the interaction of AM with sterol membranes [260]. Cholesterol and ergosterol enhance AM permeabilization, thus bringing along potent cytotoxic and antifungal capacities. In addition, AM3 showed higher affinity to ergosterol membrane; this suggests formation of a more stable complex, which may provide insights for a new antifungal drug [216]. 

Another structural class of AM related-compounds isolated from benthic *Amphidinium* species—amphirionin-5, seems to potentiate proliferation of murine bone marrow stromal ST-2 cells and murine osteblastic MC3T3-E1 cells [261]. Interestingly, another study encompassing amphirionin-4 [262] reported promotion of high intensive proliferation only in murine bone marrow stromal ST-2 cells at low concentrations, but not in NIH3T3 and MC3T3-E1 cells. It was suggested that ST-2 cells, treated with amphitionin-4, experience an increase in actin and tubulin—so enhancement of assembly of proteinaceous cytoskeleton may be involved. The proliferation-promoting ability in ST-2 cells by amphitionin-4 unfolds a great potential for bone and cartilage regeneration—as well as other organs obtained from mesenchymal stem cells, derived in turn from multipotent marrow stromal cells. Since ST-2 cells are relevant toward development of lymphocytes from bone marrow cells, this compound may improve the immune system in its ability to detect infection [262].

Another AM related-compound, also isolated from *Amphidinium* benthic species, is iriomoteolide; it revealed cytotoxic activity against human cervix adenocarcinoma HeLa cells [263].

## 3. Biotoxin and Bioactive Production

### 3.1. Dinoflagellate Bioactive Supply

Owing to a wide diversity and compound production complexity, microalgae at large—and dinoflagellates in particular, are attractive as natural sources of bioactive molecules. Remember that natural product screening continues to seek and explore a variety of chemical structures, for eventual use as structural models for new drug development by the pharmaceutical industry [265]. 

Despite its potentially wide applicability, unavailability of dinoflagellate-generated material to sufficient amounts has raised systematic challenges in attempts to further biochemical investigation and clinical testing—thus compromising eventual development into commercial products [266]. *Cripthecodinium chonii* is the only nontoxic dinoflagellate grown to commercial level as of now; it indeed produces docosahexaenoic acid (DHA) to great levels. DHA is used for enrichment of infant formulae—and its production is via heterotrophic culture in conventional fermenters [267]. Only scarce quantities of bioactives from photoautotrophic dinoflagellates are indeed commercially available (Table 2). 

Such compounds derive from a limited number of dinoflagellates (e.g., *Gambierdiscus toxicus*, *Prorocentrum concavum*, *Karenia brevis*, *Protogonyalaux* sp.) [3]—and prices can range from 1000 up to 500,000 €/mg, depending on purity and source. Furthermore, these substances are often discontinued without previous notice, and the effective purity and quantity claimed by companies is sometimes doubtful [16]. Pure compounds are needed for use as analytical standards in seafood safety screening programs, as well as research purposes (e.g., study of mechanism of action and pharmacology); lack, or unsuitable amounts of material will obviously hamper regular development of those studies [268].

Dinoflagellates in general grow slowly, and are quite shear-sensitive. It might seem paradoxal that they can cause algal blooms in Nature—yet the latter are observed only during periods of calm (and warm) waters; in fact, turbulent waves disrupt such a possibility, due to the associated high shearing. Furthermore, the rate of shearing in classical bioreactors is much higher than that prevailing in relatively still open waters. Therefore, massive production of biotoxins and other products of interest from dinoflagellate culture has proven extremely difficult [97,269]. Unlike culture of microalgae at large, the maximum biomass concentration attainable in photosynthetic cultures of a dinoflagellate remains well below 1 gram per liter [3,270]; regarding toxin production per cell, it is of the order of picogram [266]. Improvements in cultivation techniques still lag far behind practical requirements: around 150 g of a pure bioactive compound is typically needed for preclinical studies and clinical trials, so current cultivation methods will require broth medium volumes in excess of 100,000 m^3^—i.e., far above that needed by a typical antibiotic [3]. Genetic and metabolic engineering have also been attempted [271] to circumvent low productivities and enhance concentrations of target compound; however, the efforts involved are absolutely not straightforward [3]. These approaches are normally available for nondinoflagellate microalgae, and target chiefly biofuel production [272,273]. The difficulties arise from the astonishingly large and complex dinoflagellate genomes—with great amounts of introns, bearing redundant repetitive noncoding sequences [274]. Their massive genome is organized into a permanent liquid crystalline form, with a high proportion of unusual bases [275]. Furthermore, dinoflagellate genes lack recognizable promoter features, as well as common eukaryotic transcription factor binding sites [276]; and their biotoxin production capabilities apparently result from multiple independent evolutionary origins [277]—which make the identification of toxin-related genes particularly complex. Despite said difficulties, high-throughput omics technologies have been moving toward exploring toxin genes and proteins related to dinoflagellate toxin production, and are expected to provide some insights about their biosynthesis in the near future [278]. 

Chemical synthesis of dinoflagellate-derived toxins had also been tested, with more than 100 steps reported in some cases. Hence, de novo synthesis is considered as exceedingly complex—and economically unfeasible at present (except in the case of okadaic acid). Nevertheless, this process has proven useful for elucidation of structure and biological mode of action in the case of several complex bioactives of dinoflagellates (e.g., brevetoxin B [279], brevetoxin A [280], gambierol [281], gymnocin A [282], azaspiracid-1 [283], and gambieric acid A [284]). Moreover, some chemical routes from biotoxin fragments have been proposed as more efficient for some compounds [3]—such as maitotoxin [118] or yessotoxin [285]; however, more practical synthetic ways remain a challenge, unlikely to succeed in the short run.

In view of the above arguments, cultivation of dinoflagellates and extraction and purification of biotoxins (and other metabolites of interest) from closed photobioreactor seems to be the best approach to obtain significant amounts of those compounds [286]. Researchers have been investing a great deal of effort into developing dinoflagellate bioreactor controlled cultures [268,287,288,289,290], and understand what mechanisms underlie their low biomass and biotoxin/bioactive productivities [3,269,270]. A deeper know-how on dinoflagellate metabolism, cultivation and production processes is essential to rationally develop reproducible and economical systems with improved productivities—in order to permit these microalgae acquire a distinctive biotechnological role in value-added biotoxin production [291], for eventual pharmaceutical and biomedical purposes.

### 3.2. Culture of Dinoflagellates and Biotoxin Production

While culturing nondinoflagellate microalgae in large volume photobioreactor cultures is a routine practice [292,293,294], dinoflagellate culturing usually poses a number of difficulties [97]. Besides their quite low rates of growth, dinoflagellates exhibit an intricate metabolism and low biomass yields—thus resulting in low biotoxin production. Said fastidious growth may be explained by a complex nucleus, with cumulative acquisition of several prokaryotic genes throughout evolution—coupled to an inefficient *Rubisco* enzyme to distinguish CO_2_ from O_2_ [295]. 

Dinoflagellate cells exert some exceptional features compared to eukaryotic cells though. They possess a distinctive nucleus with lack of nucleosomes and histones, and chromosomes remain permanently condensed, even during mitosis [295,296]. These marine microorganisms are known to have complex circadian systems that control behavior in vivo, as they establish a vertical migration pattern according to daylight and nutrient level [297]. In general, photosynthetic dinoflagellate cells divide at the end of the dark period, and grow during the light phase (corresponding to the G1 phase of the cell cycle), precisely when production of many toxins occur. Apparently, coupling progression of cell cycle to cell growth enables them to make best use of available resources [295]. Therefore, one promptly realizes that dinoflagellate cells are extremely complex, and have singular metabolic requirements that can hardly be provided by conventional (closed) photobioreactor configurations and operating conditions. In fact, classical photobioreactors comprise simplistic modes of light supply (e.g., continuous external illumination)—which, combined with the typical uniform levels of nutrients, may break down natural rhythms and cause metabolic behavior to deviate from the original one. Continuous supply of CO_2_ is also a *sine qua non* for photosynthesis, owing to its low solubility in water—which calls for turbulence to minimize resistance to mass transfer. However, dinoflagellates are extremely sensitive to turbulence, since it leads to high shear stress or cell damage [3]. Even though dinoflagellate microalgae may grow well in natural environment (i.e., HABs), agitation and turbulent conditions in water columns in vitro have proven unsuccessful. It has been suggested that hydrodynamic forces may reduce time-integrated light exposure of individual cells, and promote physical dispersion; another explanation claims that mechano-external stimuli impact directly on cell physiology [298]. 

Nutritional requirements, illumination (i.e., cycle, intensity, irradiance) and specific patterns of agitation (i.e., laminar flow) are thus essential issues to circumvent their low rates of growth, and accordingly attain higher levels of metabolite synthesis. Parameters such as optimal temperature, pH, oxygen tolerance and ionic strength are also important, because they can influence production of some toxins. For instance, temperature and salinity were shown to induce variations in saxitoxin content in *Alexandrium catenella* [299,300]; and temperature and light have been shown to affect palytoxin-like compounds content in *Ostreopsis ovata* [301], as well as amount of okadaic acid (OA) produced by *Prorocentrum belizeanum* [302]. Elucidation of what triggers synthesis of biotoxins and other bioactive compounds, and mitigation of shear-stress through reactor engineering are central issues to be addressed regarding mass cultivation of dinoflagellates and bioactive production thereby.

#### 3.2.1. Nutritional Requirements

The composition of the nutrient mixture in growth media can strongly influence dinoflagellate survival (i.e., cell division) and biotoxin production. The L1 is the most frequently used formulation to culture dinoflagellates [303], despite the fact that it was originally developed to grow marine diatoms. Other common cited media are f/2 and K, initially designed to grow diatoms [304,305] and oligotrophic oceanic phytoplankters [306], respectively. Media formulation typically derives from enrichments of natural seawater, and is almost exclusively used in dinoflagellate culture. 

In some dinoflagellate cultures, the effect of macronutrients is essential for maintenance of cellular processes; for instance, nitrates and phosphates seem to trigger different responses regarding biotoxin synthesis. Gallardo-Rodríguez et al. [307] have shown unsatisfactory phosphorous concentrations in L1 basal medium to achieve high growth rates and biomass yields of *Protoceratium reticulatum* cultures. In addition, cell-specific production of yessotoxin bioactive was not influenced by concentration of phosphate, but by higher nitrate concentrations. On the other hand, studies with *Alexandrium* spp. and *Karenia brevis* have demonstrated that limited-phosphorous concentrations induce higher biotoxin content [308,309]. Other reports suggest an increase in toxin content when N and P are severely depleted, thus suggesting a synergistic effect of their availability [310]; while studies using *Ostreopsis ovata* suggest an opposite effect, with biotoxin production limited by N and P depletion [311]. 

Trace elements and vitamins have also proven to be of great importance. For instance, *P. reticulatum* was found to exhibit higher growth rates when selenium and iron were added to cultures, and yessotoxin production was significantly improved with selenium addition [312]. Field and culture-based studies with HAB dinoflagellates support the idea that exogenous B vitamins (i.e., B_1_, B_7_, B_12_) have the potential to broadly influence marine biomass productivity and associated composition [313]. Tests with *P. reticulatum* and *K. veneficum*, using artificial neural networks as predictive tool for nonlinear interactions among all nutrients in culture media, suggest that micronutrients and vitamins (even to lower concentrations) are relatively more significant than macronutrients toward growth of both microalgae [314,315]. 

Since medium formulations comprise many components, virtually hundreds of combinations are possible to improve biotoxin production—but quite difficult to test in practice. For that reason, the process to improve nutritional requirements of dinoflagellates may to advantage resort to a genetic-algorithm (GA). This tool is superior in performance to conventional statistical experimental designs, and has been commonly employed to develop microbial culture media [316]. GA-based stochastic search is able to explore a large experimental space, and has been successfully applied in medium formulation for *P. reticulatum* and *K. veneficum*. The new media developed have allowed 40% and 190%-enhancement of biotoxin titer, and 60% and 120%-enhancement in final cell concentration relative to basal L1 medium, respectively [317,318]. 

The effect of nutritional requirements upon overall growth and total amount of biotoxin synthesized seem to strongly depend on dinoflagellate species. Hence, efforts to find better medium formulations, via efficient methods of search and optimization, remain a priority. 

#### 3.2.2. Culture Light Provision

Light is the basic energy source, and a critical parameter for dinoflagellate autotrophic growth—and thus for achieving higher productivities. Both natural and artificial light have been reported to bring about growth of dinoflagellates of interest [270,289,302]. Artificial light provides better control of the light spectrum, irradiance or photosynthetic photon flux density (PPFD), as well as photoperiod (light/dark cycle) in closed photobioreactors. Several culture studies have employed conventional cool fluorescent lamp (Table 3), but light-emitting diodes (LEDs) are gaining importance in dinoflagellate culturing [319]. LED performance is very similar to fluorescent light, but they require less energy and narrower wavelength bands are possible. They are also less damaging to dinoflagellate cells, as they do not generate excessive heat; and can be easily designed to convey predefined levels of light delivery to photobioreactors. Until now, *K. veneficium* and *A. tamarense* were the only dinoflagellate species of interest to be cultured with success using LED technology, at pilot-scale [319,320]. For instance, *A. tamarense* growth was stimulated under blue LED, but suppressed under yellow and red LEDs to below 70 µmol_photon_.m^−2^.s^−1^. In fact, high growth efficiencies under blue wavelength have been reported for several dinoflagellates [320], but a correlation between cellular toxin levels and wavelengths remains to be established [321].

The light intensity (irradiance) and utilization efficiency are crucial in dinoflagellate cultures, and consequently in toxin bioproduction. Light energy should be delivered evenly over the photobioreactor; in order to prevent growth-limiting, photoxidation and/or photoinibition, an adequate PPFD must indeed be provided to cells [322]. Conventional green microalgae can stand elevated irradiance levels (e.g., 3000 µmol_photon_.m^−2^.s^−1^) [302], but those levels are detrimental for dinoflagellates. Several intensities have been reported for different dinoflagellate cultures [302,323,324]. Maximal and optimum intensity thresholds in dinoflagellate cultures seem to be species-dependent [325]. While some dinoflagellates grow better under low light intensities (e.g., 10–40 µmol_photon_.m^−2^.s^−1^), others can effectively grow between 50–500 µmol_photon_.m^−2^.s^−1^ or even more [47,268,324,325]. On the other hand, the light time exposure or photoperiod are also of interest for growth and biosynthesis of toxins. The most commonly used light/dark cycles are 12 h/12 h, 14 h/10 h and 16 h/8 h (Table 3). Optimal cellular DTX-1 and OA concentrations and good growth performance of *P. lima* were reported under 12 h/12 h photoperiod, thus emphasizing the importance of photosynthesis and dark respiration in such toxin biosyntheses [321]. Early studies suggests that biotoxin production of microalgal cells is controlled by light and dependent on cell cycle [326,327]. The biosynthesis of SPX in *A. ostenfeldii* is in fact governed by light-dependent mechanisms; toxin concentration per cell quota increases in the beginning of the dark period, probably corresponding to the G1 or S phase cell cycle [328]. 

Understanding the photosynthetic apparatus and light requirements at large-scale will pose great challenges in attempts to obtain successful amounts of dinoflagellate biomass and biotoxins. Several aspects regarding light have thus to be considered for closed bioreactor systems.

#### 3.2.3. Bioreactor Culture and Design

The dropping productivities associated with dinoflagellate growth in photobioreactors are often related to the shear-sensitivity of their cells—as made apparent by the negative effects of turbulence. The success of biotoxin production for pharmaceutical and/or investigational purposes implies attainment of adequate amounts of biomass in a safe way—which challenges conventional photobioreactor engineering design [286,336]. At present, cultivation in open ponds is not a viable solution for safety reasons and environmental contamination [3]. The majority of reports on dinoflagellate cultivation are indeed limited to flask or bottle cultures at laboratory scale—yet complementary studies have been developed aiming at larger scale operation [344]. Based on different technologies employed, volumes ranging from 2 L to 700 L have been tested (Table 3). The culture systems at stake include a variety of configurations—encompassing from carboys, chemostats or stirred-tanks, to typical airlift, bubble column, tubular reactor and flat-plate PBRs (photobioreactors). The technology Twin-Layer PBR, at laboratory scale, introduced by Benstein and coworkers [341] for growth of *Symbiodinium voratum* and production of peridinin pigment in an immobilized support, suggested a new configuration approach that could also be suitable for biotoxin production. 

Culture operation modes can greatly influence the efficacy of biomass and toxin productivities. Beuzenberg and coworkers [268] have demonstrated that *K. selliformis*, *A. ostenfeldii* and *K. brevisulata* continous cultures in column PBRs led to substantial improvements in productivity. Biotoxin yields increased 2–3 fold relative to batch mode, except for *K. brevisulata*. Fuentes and co-workers [289] reported successful production of biomass by *A. caratarea* in a semi-batch mode, either indoors or outdoors. Wang and coworkers [333,334] observed lower growth of *A. tamarense* under semi-batch mode—possibly due to inadequate dilution cycles and excess disturbance on cultures; however, C2 toxin yields were considerably higher in batch mode. Special attention should also be given to aeration regime applied to dinoflagellate cultures. Despite turbulent environments triggering a few negative effects upon those cultures—e.g., reactive oxygen species accumulation, lipoperoxide formation, changes in cell membrane fluidity, or/and calcium mobilization [3,269], aeration seems to improve biomass productivities and biotoxin concentrations if provided and carefully controlled [336]. Air flow helps stripe dissolved oxygen, hence minimizing harmful effects upon microalga cells [270]. Hu and co-workers [332] reported on a two-step batch culture method, first favoring growth in static conditions, and then applying aeration in a subsequent step to improve saxitoxin yields by *A. tamarense*. Wang and co-workers [333,334] have reached higher contents of C2 toxin *Alexandrium* permeabilization following a similar strategy; Gallardo-Rodríguez and co-workers [269,286] also observed increasing levels of yessotoxin in cultures of *P. reticulatum*. 

Consequently, bioreactor scale-up remains largely undeveloped, so researchers are to explore several economical and viable options regarding bioreactor design and culture strategies. Successful scale-up will ultimately dictate industrial feasibility of any process based on dinoflagellate biotoxins.

## 4. Final Considerations

Dinoflagellates have proven to be a rich biotechnological source of biotoxins, with interesting biological activities that are potentially useful in a wide spectrum of pharmacological and medical fields, besides being promising tools for chemical biology. Despite such recognized value, scarcity of such biotoxins for preclinical testing (and later for commercial exploitation) remains a major issue. As chemical synthesis and genetic engineering are extremely difficult to achieve, a lot of effort and resources have been directed to improve modes of culturing dinoflagellates in photobioreactors, aimed at obtaining larger biotoxin concentrations. Nutritional and operation conditions, such as light and aeration/agitation patterns, have to take into account that dinoflagellates obey specific circadian rhythms, and are extremely sensitive to shearing when cultivated in a reactor. Conventional engineering and bioreactor design methods have thus to be overcome, so as to circumvent the fastidious growth and shear-sensitivity of dinoflagellate cells.

## Figures and Tables

**Table 1:** Selected patents related to biotoxins produced by dinoflagellates and potential therapeutic uses.

Patent Name/Application	Biotoxin Used	Year	Reference
10′,11′-modified saxitoxins useful for treatment of pain	Modified saxitoxin	2017	US20170029431
Use of sodium channel blockers for treatment of neuropathic pain developing as consequence of chemotherapy	Tetrodotoxin, saxitoxin (analogues and derivatives)	2017	US20170000797
Ladder-frame polyether conjugates	Brevetoxin, maitotoxin, yessotoxin, gambierol	2016	US20160128321
Sodium channel blocker for treatment of loss of superficial sensitivity	Gonyautoxin	2016	US20160000793
Palytoxyn, medical use and process for its isolation	Palytoxin	2014	EP3087172
Neosaxitoxin combination formulations for prolonged local anesthesia	Neosaxitoxin	2014	WO2014145580
Treatment of loss of sense of touch with saxitoxin derivatives	Saxitoxin	2014	EP2533785
Using yessotoxin and its derivatives for treatment of gliomas	Yessotoxin and derivatives	2013	ES2393696
Use of yessotoxin and analogues and derivatives thereof for treating and/or preventing neurodegenerative diseases linked to tau and beta amyloid	Yessotoxin (analogues and derivatives)	2013	US20130035302
Use of gambierol for treating and/or preventing neurodegenerative diseases related to tau and beta-amyloid	Gambierol	2012	US20120283321
Use of yessotoxins and derivatives thereof for treatment and/or prevention of metabolic diseases	Yessotoxin and derivatives	2012	WO2012140298
Use of gymnodimine, analogues and derivatives for treatment and/or prevention of neurodegenerative diseases associated with tau and beta-amyloid	Gymnodimine (analogues and derivatives)	2012	US20120245223
Therapeutic use of yessotoxin as human tumor cell growth inhibitor	Yessotoxin	2011	EP1875906
Methods and compositions for studying, imaging, and treating pain	Saxitoxin, gonyautoxin and other analogues	2011	WO2010129864
Use of sodium ion channel blocker in treating biology drug resistance of antibiotic	Tetrodotoxin, saxitoxin	2009	CN101450056
Polyether brevetoxin derivatives as treatment for cystic fibrosis, mucociliary dysfunction, and pulmonary diseases	Brevetoxins and derivatives	2008	US7399782
Use of sodium channel blockers for treatment of preterm labor	Tetrodotoxin, saxitoxin (analogues and derivatives)	2007	WO2007096170
Polyether brevetoxin derivatives as treatment for neurotoxic shellfish poisoning and ciguatera fish poisoning	Brevetoxin and derivatives	2005	WO2005027903
Methods of treating wounds with gonyautoxins	Gonyautoxin	2005	WO2005110275
Dinoflagellate karlotoxins, methods of isolation and uses thereof	Karlotoxin	2005	US2005/0209104
Use of yessotoxin in treatment of allergic and asthmatic processes	Yessotoxin	2003	EP1875907

**Table 2:** Commercially available biotoxins—with corresponding suppliers, sources and price range (per mg).

Toxin	Supplier	Source	Price Range (€ mg^−1^)
Okadaic acid	SGA GEN LCL MER SL TC BP SCB	*Prorocentrum concavum* unkown unkown *Prorocentrum* sp. unkown unkown unkown *Prorocentrum* sp.	1430 3510 561 2538 3479 5000 1210 1850
Okadaic acid, ammonium salt	SGA LCL	*Prorocentrum concavum* unkown	16,620 561
Okadaic acid, sodium salt	SGA GEN LCL SL	*Prorocentrum concavum* unkown unkown unkown	9730 2320 561 2657
Okadaic acid, potassium salt	SGA GEN LCL	*Prorocentrum concavum* unkown unkown	8050 2320 561
Okadaic acid, solution	MER	*Prorocentrum* sp.	4480
Tetrodotoxin	GEN TC	unkown unkown	451 195
Saxitoxin, diacetate	GEN	unkown	9920
Brevetoxin 2	GEN	unkown	17,960
	MER	*Karenia brevis*	4390
	SCB	*Karenia brevis*	5970
Brevetoxin 3	GEN	unkown	17,530
	MER	*Karenia brevis*	3880
Brevetoxin 9	GEN	unkown	19,380
Yessotoxin, antibody	GEN	unkown	1580
Maitotoxin	LCL	unkown	unkown
Azaspiracid-1	SCB	Marine mussel	357,000
Azaspiracid-2	SCB	Marine mussel	359,000
Azaspiracid-3	SCB	Marine mussel	540,000

SGA: Sigma-Aldrich (St. Louis, MO, USA) (www.sigmaaldrich.com); GEN: Gentaur Molecular Products (Kampenhout, Belgium) (www.gentaur.com); LCL: LC Laboratories (Woburn, MA, USA) (https://www.lclabs.com/); WK: Wako Pure Chemical Industries Ltd. (Osaka, Japan) (www.wako-chem.com); MER: Merck Millipore; SL: ScienceLab (Dickinson, ND, USA) (www.sciencelab.com); TK: Tocris (Bristol, UK) (www.tocris.com); Bertin Pharma (Montigny le Bretonneux, France) (www.bertinpharma.com); SCB: Santa Cruz Biotecnology, Inc. (Dallas, TX, USA) (https://www.scbt.com/scbt/home/).

**Table 3:** Major results of studies on bioreactor type, culture mode and other operational conditions for dinoflagellate growth and/or biotoxin synthesis optimization.

Species and Strain	Type of Reactor	Reactor Operation	Reactor Size	Type of Aeration/Agitation	T (°C)	Irradiance Culture System	Light Intensity (µmol m^−2^ s^−1^)	Light Regime	Biotoxin Produced	Specific r-Emsarks	Ref.
*Prorocentrum lima* spp.	Carboy	Batch	36 L	Stirring (discontinuous)	18 ± 1	-	90 ± 5	Continuous dark/14 h:10 h	Okadaic acid Dinophysistoxin	-	[326]
*Protoceratium reticulatum* CAWD129	Carboy	Batch	226 L	-	-	-	-	12 h/12 h	Yessotoxin Furanoyessotoxin	Serial bulk culture (size ~14 L each)	[329]
*Azadinium spinosum* 3D9	Chemostat in series	Continuous	100 L	Stirring (not specified)	18	-	200	16 h/8 h	Azaspiracid	-	[330]
*Azadinium spinosum* 3D9	Tubular PBRs in series	Continuous	100 L	Stirring (Rushton turbine)	18	Neon tube lamps	200	16 h/8 h	Azaspiracid	Culture collected in an aerated harvesting tank (300 L)	[331]
*Alexandrium tamarense* ATHK	Airlift PBR	Batch	2.5 L	Airlift	22	Cool-white fluorescent lamps	60	Continuous	Saxitoxin	Two-step batch culture method	[332]
*Alexandrium tamarense* ATCI01	Glass rectangular tank	Batch/Semi-continuous	70 L 40 L	Air bubbling (continuous)	23	Cool-white fluorescent lamps	108	16 h/8 h	C2 toxin	-	[333] [334]
*Protoceratium reticulatum* GG1AM	Stirred-PBR	Batch/Fed-batch /Semi-continuous	15 L	Stirring (impeller)/gas sparging	19 ± 1	Cool-white fluorescent lamps	242–766	-	Yessotoxin	-	[290]
*Protoceratium reticulatum* GG1AM and VGO764	Stirred-glass fermenter	Semi-continuous Fed-batch Continuous	2 L	Stirring (impeller)	-	Cool-white fluorescent lamps	(e.g., 34, 44, 54, 66, 81, 99, 100)	-	Yessotoxin	-	[335] [270]
*Amphidinium carterae* ACRN03	Airlift bubble column PBR	Semi-continuous	540 L 320 L 48 L	Compressed air (continuous)	23.3 ± 2.2	-	158 ± 22 (indoor) ~464 (outdoor)	18 h/ 6h	N/A	Production of biomass	[289]
*Karenia selliformes* CAWD79 *Alexandrium ostenfeldii* CAWD135 *Karenia Brevisulcata* CAWD82	Column PBRs Carboy	Batch/ Continuous	5.4 L 52 L	Magnetic stirring/Air bubbling (low)	-	Cool-white fluorescent lamps	38	12 h/12 h	Gymnodimine Spirolide Brevisulcatic acid	-	[268]
*Karlodinium veneficum* K10	Bubble column PBR	Batch (sequential)	80 L	Gas Sparging	21 ± 1	LEDs	220 1500 (>7th day)	12 h/12 h	N/A	-	[287]
*Amphidinium* sp.	Flat-bottom flask	Batch	3 L	-	25	-	108	16 h/8 h	Amphidinolide	-	[248]
*Alexandrium minutum* AMAD06 and AMAD16	Alveolar panel PBR	Batch/Semi-continuous	4 L	Alveoli	-	Cool-white fluorescent lamps	100	12 h/12 h	Gonyautoxin/fraction of saxitoxin, neosaxitoxin and C-toxin	-	[336]
*Alexandrium ostenfeldii* CCMP1773	Flat-bottom vessel	Batch	8 L	N/A	16	Cool-white fluorescent lamps	155	12 h/12 h	Spirolide	-	[337]
*Alexandrium ostenfeldii* CCMP1773	Column PBR	Continuous	100 L	Stirring (paddle impeller turbines)	18	Cool-white fluorescent lamps	190	16 h/8 h	Spirolide	-	[337]
*Alexandrium pacificum* HYM9704	Chemostat system	Batch Semi-continuous	2 L	Air bubbling	15	Cool-white fluorescent lamps	150	12/12	C-toxin Gonyatoxin Saxitoxin	-	[338]
*Amphidinium carterae* (Hulbert)	Plastic cylinder	Batch	40 L	Airlift	22	Cool-white fluorescent lamps	40	12 h/12 h	N/A	Identification of pharmacological activity in vitro using algal extracts	[339]
*Prorocentrum lima* CCMP 2579	Vertical flat PBR	Batch	100 L	Air bubbling	20	Cool-white fluorescent lamps	100	12 h/12 h	Okadaic acid Dinophysistoxins	-	[321]
*Karlodinium veneficum* ICMB 252 *Alexandrium. minutum* AMP4	Column PBR	Batch (indoors) Semi-continuous (outdoors)	350 L	Air bubbling	20 ± 1 (indoors) Variable (outdoors)	Cool-white fluorescent lamps	110 (indoors) 202–4020 (outdoors)	12 h/12 h	N/A	Each PBR column size ~35 L each Production of lipids	[340]
*Symbiodinium voratum*	Twin Layer PBR (Biofilm-immobilization)	Continuous	414 cm^2^	Air tube (continuous)	23 ± 1 25 ± 3	Cool-white fluorescent and Sodium lamps	26 ± 2 73.5 ± 17.5	14 h/10 h	N/A	Production of peridin	[341]
*Amphidinium carterae* (JHWAC) *Symbiodinium* sp. (JHLSD1) *Prorocentrum rathymum* (JHWPMX1)	Vertical column PBR system (12× column)	Batch	700 L	Air bubbling	20 ± 1	Cool-white fluorescent lamps	40–50	12 h/12 h	N/A	Each PBR column size ~60 L each Screening antioxidant properties	[342]
*Karlodinium veneficum* (CCMP 2936)	Vertical column PBR	Batch	31 L	N/A	24	LEDs	50	14 h/10 h	N/A	Study on vertical migration	[343]

N/A—Not Applicable.
